# The Hygiene of Sewing-Machines

**Published:** 1858-06

**Authors:** 


### THE HYGIENE OF SEWING-MACHINES

WAS very aptly illustrated to-day, by the casual remark of a lady of education and energy, which is worth more than a dozen purposely penned paragraphs for publication. "After using WHEELER & WILSON'S sewing-machine for several hours, I feel an unusual freshness and vigor, and have a relish for dinner, which of itself is delightful. I account for it from the fact, that both mind and body are called into active exercise; nearly every muscle of the system is brought into gentle play, while a high mental exhilaration is experienced from that interested attention which the successful management of one requires; at the same time, the insufferable monotony of the needle is avoided, and the necessity is removed of that painful straining of the eye, which prematurely impairs the vision, and makes glasses necessary many years before their time."

